# Heavy burden of intestinal parasite infections in Kalena Rongo village, a rural area in South West Sumba, eastern part of Indonesia: a cross sectional study

**DOI:** 10.1186/s12889-015-2619-z

**Published:** 2015-12-24

**Authors:** Saleha Sungkar, Anggi P. N. Pohan, Antari Ramadani, Nafisah Albar, Fitri Azizah, Antonius R. A. Nugraha, Aprilianto E. Wiria

**Affiliations:** Department of Parasitology, Faculty of Medicine Universitas Indonesia, Jakarta, Indonesia; Department of Parasitology, Leiden University Medical Center, Leiden, The Netherlands

**Keywords:** Intestinal parasitic infection, Helminthiasis, Hygiene, Intestinal protozoa, Indonesia

## Abstract

**Background:**

Intestinal parasitic infections (IPIs) are one of the major public health problems, especially in the rural area of developing countries with low socio-economic status and poor sanitation. The study was aimed to determine the prevalence of IPIs among the inhabitants of a rural area in South West Sumba, eastern part of Indonesia.

**Methods:**

A cross-sectional study was done in Kalena Rongo village, South West Sumba in April 2014. Stool samples were collected and examined for IPIs using direct smear method.

**Results:**

Faecal samples were collected from 424 of 473 inhabitants of the village, age 2 months to 80 years. About 95.5 % (405/424) of the participants had any IPIs. The most prevalent parasites found were *Ascaris lumbricoides* 65.8 % (279/424), *Trichuris trichiura* 60.4 % (256/424), hookworms 53.5 % (227/424), *Blastocystis hominis* 34.4 % (146/424), *Entamoeba histolytica* 17.9 % (76/424), and *Giardia lamblia* 4.5 % (19/424). The villagers used no latrine and defecated in their backyard. Clean water sources were scarce and far from the village.

**Conclusions:**

In Kalena Rongo village, the rural area in eastern part of Indonesia, the finding of IPIs was conspicuous and therefore expressed the poor hygiene and absence of proper sanitation in the area. Integrated efforts, such as improving infrastructure to provide clean water source and educating the inhabitants for appropriate hygienic lifestyle are needed.

## Background

Intestinal parasitic infections (IPIs), which can be caused by infection with soil-transmitted helminths (STH) and intestinal protozoa, is a public health problem [1–3]. The elimination of morbidity due to helminth infections in children has been aimed to be achieved by 2020, whereas periodic deworming program has been endorsed for decades, especially in 2001 during World Health Assembly and in 2009 during the Special Programme for Research and Training in Tropical Diseases [1, 4]. One of the major target for the elimination is the STH (*Ascaris lumbricoides*, hookworms (*Necator americanus*, *Ancylostoma duodenale*), and *Trichuris trichiura*) as being the most prevalent helminth group globally [5]. The deworming program can be easily integrated with child health days program or with supplementation programs for preschool children or with school health programs was targeted to be successfully obtained, by treating regularly at least 75 % of the children living in endemic areas (an estimated 873 million). By the year 2013, the coverage has reached for over 368 million school children, and therefore, more than 50 % children are still at risk globally [1]. Furthermore, the intestinal protozoa such as *Entamoeba histolytica*, *Giardia lamblia* and *Cryptosporidium spp*. can be easily found infecting individuals who have already harboured STH. This co-infection can cause major impact in children, pregnant women and in HIV-infected patients [6]. STH and intestinal protozoa infections cause IPIs and have been linked with poor environmental sanitation, poor personal hygiene, and low educational level [7]. Approximately 200 million people in South East Asia (SEA) are living under poverty line, on less than US$2 per day, residing in poorly infrastructure environment and becoming more vulnerable to such infections, known as neglected tropical diseases (NTDs) [3]. Indeed, improved infrastructure, better sanitation and deworming program have reduced IPIs in the urban area of developing countries, but the situation in their rural part is largely unknown [8].

Indonesia is the largest country with the largest economic power in SEA; nevertheless the country contributes to more than half of the bottom 200 million poorest of SEA citizen [3]. East Nusa Tenggara is the third poorest province in Indonesia and has high prevalence of IPIs [9]. Moreover, Southwest Sumba, a recently segregated district from West Sumba, is the second poorest district in East Nusa Tenggara and has the highest number of uneducated population (57.3 %), household without latrine access (52.4 %), as well as having unprotected water springs as the main water source (44.4 %) [10]. According to a survey by Ministry of Health in 2011, West Sumba had 29.5 % STH prevalence [11]. However, the number might be underestimated as villages in South West Sumba, had very limited access to clean water and the use of latrine was rare, and most likely the survey was not reached the remote areas. The aim of this study was to determine the prevalence of IPIs in this area.

## Methods

### Study population

South West Sumba consists of 11 districts. North Kodi is the most populated and the largest district with 51.417 populations, total area of 235,7 km2 and has a border with the Indian Ocean in its west. North Kodi has the lowest average of the rainfall rate among the districts in Southwest Sumba with the rate of 161,5 mm per year and the average of rainy days is only 8 days yearly. Around 44 % population of North Kodi had never been in formal education even though the compulsory education program in Indonesia is 9 years [12].

Sumbanese traditional houses consist of 3 levels story: the livestock pen, the main floor, and the storage tower which is made of thatched roof. Another part of the house is the veranda, a raised bamboo platform which spans the entire front of the house. The livestock such as pigs, poultry, and dogs are kept underneath the main floor. Even though sheds are prepared outside for horses, water buffaloes, and cows, the animals casually defecate around the house [12].

Kalena Rongo, one of the villages in North Kodi, with total inhabitants 473 people, is a typical rural village of remote area in eastern part of Indonesia. Access to the area was difficult. The only main road is partially made of tarmac and the smaller roads are gravel. For food source, people mainly relied on wild plants, such as corn and sweet potatoes, and rearing livestock such as cows, goats, pigs, and poultry. The farming methods are traditional by using human power or with the help of Sumba horses and water buffaloes. The village was chosen for this study because it was the poorest village in the region, had a limited access to clean water and therefore had poor hygiene and sanitation. We aimed to reach all of the population in the village, and therefore no sample size was calculated.

### Study design

This cross-sectional study was done in Kalena Rongo, South West Sumba, East Nusa Tenggara. Data was collected in April 2014 by the researchers from the Department of Parasitology, Faculty of Medicine Universitas Indonesia (FMUI) as part of FMUI community engagement program.

### Intestinal parasite examination

The stool samples were collected early in the morning and preserved using formalin 5 % in order to be transported and examined in the Department of Parasitology FMUI, Jakarta. The examination was done four days after the collection with a direct smear method. A small amount of stool was smeared (1–2 mm^3^) on a glass slide, mixed with 1 % lugol reagent, covered with a cover slip and examined under a binocular optical microscope (Olympus CX21, Olympus America Inc., USA), first with low power (10x) magnification followed by reading with high dry objectives (40x). The procedure was done three times, where each slide was read by two technicians (Wulan and Pariyah) from Department of Parasitology FMUI. Inconsistent results were re-read concomitantly by both technicians. Findings of eggs for helminths and cysts for protozoa were considered positive results.

### Statistical analysis

Pearson chi-squared test was used to analyse the association between the IPIs and age and sex. Participants were grouped into three age groups of <5 years, 5–12 years, and >12 years. This a priori clustering reflects the environment experienced by subjects, e.g., predominantly inside the house for the pre-school age (<5 years), surrounding the house for grade-schooler (5–12 years), farmland and bushes for villagers working in the farm and plantation (>12 years). The 95 % Confident interval was calculated with *t* test. *P* value < 0.05 is considered significant. Statistical analysis was performed using IBM SPSS Statistics 20.0.

### Ethics statement

This study was approved by the Ethics Committee of the FMUI, ref:207/UN2.F1/etik. Informed consent was obtained from all participants, where parent/guardian consent was obtained for minors to participate in the study. Laboratory results were kept confidential. Participants with positive results for any helminth infections who were older than 2 years, were treated with single dose albendazole 400 mg. Children between 12 and 24 months received single dose albendazole 200 mg. Participants who had amoebiasis and giardiasis were referred to local health care centre to be treated according to national guidelines.

## Results

About 90 % (424/473) of the inhabitants were participated in the study where 45.5 % (193) of the participants were male vs 54.5 % (231) female. The youngest participant was 2 months and the oldest was 80 years, with the median age was 12.5 years (Table 1). We found that 92.2 % (391) of the participants were infected with at least one helminth species. The most prevalent helminth species in our study area were *A. lumbricoides* (65.8 %), *T. trichiura* (60.4 %), and hookworm (53.5 %). Other helminths such as *Taenia sp.* (0.7 %), *H. nana* (0.5 %) and *H. diminuta* (0.2 %) were also found at a very low percentage. Intestinal protozoa infections were found in 44.1 % (187) participants; *Blastocystis hominis*, *E. histolytica*, *G. lamblia* and *Iodamoeba butchlii* were found in 34.4% (146), 17.9 % (76), 4.5 % (19), and 1.7 % (7) individuals, respectively (Table 1). Overall, 95.5 % of the participants were infected with at least one parasite. Most of the participants were infected by more than one species of parasite, only about 20 % (83/405) of the infected individuals had a single infection.

**Table 1:** Age and sex distribution, and intestinal parasitic infections prevalence

	Total Population	Age groups (year)			*P* value
	*n* = 424	<5	5-12	>12	
		*n* = 76	*n* = 136	*n* = 212	
Age (years, median, min-max)	12.5 (0.2-80.0)	2.0 (0.2-4.0)	8.0 (5.0-12.0)	30.0 (12.0-80.0)	**-**
Sex (female, %, 95 % CI)	231 (54.5, 49.7-59.2)	36 (47.4, 35.9-58.9)	71 (52.2, 43.7-60.7)	124 (58.5, 51.8-65.2)	**-**
Any helminth infections (n, %, 95 % CI)	391 (92.2, 89.7-94.8)	64 (84.2, 75.8-92.6)	134 (98.5, 95.3-1.00)	194 (91.5, 87.7-95.3)	**0.002**
*Ascaris lumbricoides* (n, %, 95 % CI)	279 (65.8, 61.2-70.3)	44 (57.9, 46.5-69.3)	98 (72.1, 64.4-79.7)	137 (64.6, 58.1-71.1)	0.10
Hookworm (n, %, 95 % CI)	227 (53.5, 48.8-58.3)	27 (35.5, 24.5-46.5)	87 (64.0, 55.8-72.1)	113 (53.3, 46.5-60.1)	**<0.001**
*Trichuris trichiura* (n, %, 95 % CI)	256 (60.4, 55.7-65.1)	36 (47.4, 35.9-58.9)	99 (72.8, 65.2-80.4)	121 (57.1, 50.4-63.8)	**0.001**
*Taenia sp.* (n, %, 95 % CI)	3 (0.7, 0.1-1.5)	0 (0)	0 (0)	3 (1.4, 0.0-3.0)	0.22
*Hymenolepis nana* (n, %, 95 % CI)	2 (0.5, 0.1-1.1)	0 (0)	0 (0)	2 (0.9, 0.0-2.3)	0.37
*Hymenolepis diminuta* (n, %, 95 % CI)	2 (0.2, 0.2-0.7)	1 (1.3, 0.0-3.9)	0 (0)	0 (0)	0.10
Any protozoa infections (n, %, 95 % CI)	187 (44.1, 39.4-48.9)	21 (27.6, 17.4-37.9)	64 (47.1, 38.6-55.6)	102 (48.1, 41.3-54.9)	**0.006**
*Blastocytis hominis* (n, %, 95 % CI)	146 (34.4, 29.9-39.0)	17 (22.4, 12.8-32.0)	53 (39.0, 30.7-47.3)	76 (35.8, 29.3-42.4)	**0.042**
*Entamoeba histolytica* (n, %, 95 % CI)	76 (17.9, 14.3-21.6)	6 (7.9, 1.7-14.1)	26 (19.1, 12.4-25.8)	44 (20.8, 15.3-26.3)	**0.039**
*Giardia lamblia* (n, %, 95 % CI)	19 (4.5, 2.5-6.5)	4 (5.3, 0.1-10.4)	11 (8.1, 3.5-12.7)	4 (1.9, 0.4-3.7)	**0.023**
*Iodamoeba butchlii* (n, %, 95 % CI)	7 (1.7, 0.4-2.9)	1 (1.3, 0.0-3.9	4 (2.9, 0.1-5.8)	2 (0.9, 0.0-2.3)	0.35
Any infections (n, %, 95 % CI)	405 (95.5, 93.5-97.5)	66 (86.8, 79.1-94.6)	135 (99.3, 97.8-100)	204 (96.2, 93.6-98.8)	**<0.001**
1 infections (n, %, 95 % CI)	83 (19.6, 15.8-23.4)	24 (31.6, 20.9-42.3)	16 (11.8, 6.3-17.3)	43 (20.3, 14.8-25.7)	**-**
2 infections (n, %, 95 % CI)	132 (31.1, 26.7-35.6)	24 (31.6, 20.9-42.3)	41 (30.1, 22.3-38.0)	67 (31.6, 25.3-37.9)	**-**
3 infections (n, %, 95 % CI)	116 (27.4, 23.1-31.6)	12 (15.8, 7.4-24.2)	47 (34.6, 26.5-42.7)	57 (26.9, 20.9-32.9)	**-**
>3 infections (n, %, 95 % CI)	74 (17.5, 13.8-21.1)	6 (7.9, 1.7-14.1)	31 (22.8, 15.7-29.9)	37 (17.4, 12.3-22.6)	**-**

95 % Confident interval was calculated with student *t* test. *P* value was calculated with Pearson chi-square, degree of freedom = 2. *P* < 0.05 is considered significant

Percentage of female (97.4 %) infected with at least one parasite was slightly higher than male (93.3 %) with *p* value = 0.04 (*Χ*^2^, degree of freedom (df) = 1). Female showed higher intestinal protozoa infections (49.8 %) than male (37.3 %) with *p* value = 0.01 (*Χ*^2^, df = 1). There was a slightly higher percentage of helminth infection in female (93.9 %) compared to male (90.2%) however it was not statistically significant (*Χ*^2^, df = 1, *p* = 0.15).

The youngest helminth infected individual was 5 months, while the youngest individual infected by intestinal protozoa was 2 years old. The oldest villagers being infected with helminths was 80 years old and the oldest infected with protozoa was 75 years old. The infection status within three age groups (<5 years, 5–12 years, and >12 years) according to the possible risk for environmental exposure is presented in Table 1. The highest percentage of helminth infected individuals was found among those who were 5–12 years old. About 90 % of this group had polyparasitism.

## Discussion

The IPIs in Kalena Rongo were found in almost all villagers from the very young age to the elderly. Among the infected individuals, approximately 80 % had polyparasitism. Direct examination method of the formalin preserved samples was used as the diagnostic method. It was clear that intestinal parasites were highly endemic in the area.

Kalena Rongo has low rainfall rate which often dries out the only river in area, located 5–8 km from the village. The government had made several wells but the number was too few to fulfil the needs of the villagers. Due to the lack of water resources, villagers often did not wash their hands before eating and only showered once a week in a public well, in which the same water was also used for drinking and cooking. Those explain the poor hygiene behaviours. The villagers shared the same lifestyle. From the collection of personal information regarding details hygiene behaviour, everyone had no latrine, were not washing their hand before eating, did not clean or wash after defecation, and spits randomly. Therefore, we could not analyses the association statistically. In addition, there were no public latrine nor toilet facilities. Therefore, open defecation was inevitable and after defecation villagers wiped their anal area using dispersed leaves or as mentioned before, they were not cleaning up at all. It was very common for men to climb three and defecate on the three. The reason was to make their gluteus widely open and to prevent the contact with their faeces and therefore could have their gluteus “clean”.

Regarding the intestinal protozoa, reports from urban slum area have shown that contaminated water would be the infection source of the protozoa such as *Giardia sp.* and *Entamoeba sp.* [13, 14]. However, water contamination in rural settings may be more related to a higher prevalence of STH infection [7, 15]. We found that almost half of the participants were infested by the protozoa, with the youngest infected with *Giardia sp.* was 2 years old, and the one infected with *I. butchlii* (a common protozoa living in pigs’ intestine) was 4 years old. Infestation by non-pathogenic protozoa should be treated cautiously in people with malnutrition or HIV/AIDS, which may suffer from immunodeficiency. East Nusa Tenggara is the third province with the highest findings of malnourished children in Indonesia. In 2007, it has been reported that 33.6 % children under 5 years old in the region suffered malnutrition [16]. Unfortunately we did not record the nutritional status as well as health symptoms from the participants; those are also limitation of this study.

The most common species found in our study was *A. lumbricoides* (66 %)*.* Environmental factors contributed to the development and survival of eggs and larvae of *A. lumbricoides* [17]. Earthen floor increases the persistence of *A. lumbricoides* eggs for months and up to 15 years after being excreted from infected individuals. *T. trichiura* which shared similar infection pathway to *A. lumbricoides* [17] was also highly prevalent. Poor hygienic practices of not washing hands prior to eating could acquire infectious parasitic eggs from the contaminated soil and food easily.

Our result is similar with a study conducted in the rural area of Malaysia by Al-Delaimy et al. [18] whereas 98.4 % of children in that study were found to be infected by at least one parasite species and 71.4 % had polyparasitism. Another study by Garbossa et al. [13] in Argentina, showed that the prevalence of IPIs was 83.3 % and nearly 54 % of them harboured two or more parasites, with the most common finding was caused by two parasites (29 %). In a study on Flores, an island in the same region, helminth infection was harboured by more than 90 % of the population [9]. Indeed, high prevalence of IPIs can be associated with numerous factors. The possibility and the similarity of our studies with the others were most likely related to the use of unsafe water supplies as the source for drinking water, absence of a toilet in the house and defecation in indiscriminate places such as bush or river, leaving their feet unprotected during outdoor activities, and poor of personal hygiene such as not washing hands before eating [7]. The women did not wash their hands before preparing food and during feeding their children. The finding of T. trichiura eggs in the stool of 5 month olds baby is due to the feeding method conducted by the mothers. Babies had their first food (soften banana) since one month old or fed by food/rice that was first chewed by their mother. These indicate that the study area was heavily contaminated with the parasites and as the consequences, these could lead to higher rate of re-infection.

*Taenia sp.* eggs was found in the stool of 3 villagers. Taenia’s species was not determined. The findings of Taenia’s eggs indicated the close relationship between human and pig’s faeces. The swines were roamed in the surroundings and excreted around the houses. Under cooked or raw pork meat consumption was commonly found during the traditional ceremony, with poor hygienic serving. These poor behavioral practices could increase the risk of acquiring Taeniasis and in extreme case may cause neurocysticercosis [19]. The less commonly found parasites were *H. nana* and *H. diminuta*, which are usually found in rats. The *Hymenolepis sp.* eggs will not survive in warm and dry condition but if they were ingested by insects such as fleas they could survive longer [20]. It implies to the poor sanitation because the soil was contaminated with parasite eggs from human waste or food supplies were contaminated with fleas, then eaten by human. As *H. nana* is one of a few parasites that can cause autoinfection and can persist for years, this also can be a potential problem to highly immunosuppressed hosts [21].

The effort to stop the chain of IPIs transmission in the area might encounter challenges, especially due to the highly contaminated environment. WHO suggested a selective helminth treatment for area with less than 20 % of helminth prevalence, a yearly single dose Albendazole treatment when the prevalence is 20-50 % and biannually when it is above 50 %, which could be done through schools [1, 11]. Therefore, a minimum of twice a year mass treatment should be done in the region. A combination with other NTDs control, such as lymphatic filariasis (LF) annual treatment may be beneficial [3, 22]. However, deworming at school level, might be not successful as many of the villagers were not attending school. Such intensive deworming program also will not be effective without the improvement of sanitation as re-infection may occur easily [8, 9]. Multiyear mass drug treatment in combination with LF control [22] or an intensive three-month period of single dose Albendazole treatment [9] could reduce but not able to eliminate helminth infection. A low efficacy of single dose albendazole to *T. trichiura* or a possible drug resistant is another concern [23]. The people of Kalena Rongo need to be educated about hygienic lifestyle. Unfortunately under the circumstances, it is difficult for the people to accomplish good hygienic behaviour due to the water shortage. Specific interventions are needed for instance building a good quality wells for clean water reservoir and improving sanitation facilities [8]. Immediate actions by providing early interventions are required to overcome the problems such as making temporary toilets and digging holes to conceal human’s excrement that could reduce the contamination. Therefore, it is essential for the local government, health-care officials, and the villagers to work together in solving the health problem and poverty in Kalena Rongo.

Despite to the problems mentioned above, the researchers acknowledged the limitations in the method. This study had to rely on a single faecal sample instead of the ideal three consecutive samples taken for three days due to the limitation of sources. The findings of IPIs might be affected by the temporal variation in egg excretion over hours and days. Nevertheless, the infection rate seems to be overall high in general even though we did not quantify the intensity of infection. Also, to detect hookworm infections, Kato Katz or Harada Mori methods are superior, but they need fresh stool samples [24]. This led to underestimated hookworm findings. Moreover, *S. stercoralis* infection might be underestimated as well. In the study in Flores, *S. stercoralis* was reported only after PCR examination [9]. Overall, examination of samples under microscope is operator dependent, many of the intestinal parasite species might be unidentified, or misinterpreted in mild infections when only a single stool sample is analysed [24, 25]. However, direct smear microscopy method can be simple and practical and still reliable in the situation with limited resource. We had tried to improve the reliability of the diagnosis by conducting three times measurements by two experienced technicians for each samples.

Heavy and chronic STH infections can lead to anaemia, malnutrition, impairments in physical, intellectual, and cognitive development especially in children and pregnant women [17]. Also, intestinal protozoa can cause gastrointestinal disorder [26]. While researchers questioning whether having helminth infection in a certain level might be useful [27–29], there are urgent needs to expand surveillance activities for the risk for IPIs, as well as other NTDs [3]. After that, an appropriate approach should be done to ensure infection is under control to avoid morbidity in the vulnerable group.

## Conclusions

The burden of IPIs in Kalena Rongo village was very high and polyparasitism was common. Mass treatment, provision of clean water sources, sanitation facilities, and public awareness about hygienic lifestyle are required to reduce the number of IPIs.
